# Presence of *Malassezia* Hyphae Is Correlated with Pathogenesis of Seborrheic Dermatitis

**DOI:** 10.1128/spectrum.01169-21

**Published:** 2022-01-12

**Authors:** Juanjuan Li, Yahui Feng, Chen Liu, Zhiya Yang, Sybren de Hoog, Yuying Qu, Biao Chen, Dongmei Li, Huabao Xiong, Dongmei Shi

**Affiliations:** a Department of Clinical Medicine, Jining Medical University, Jining, Shandong, China; b Laboratory of Medical Mycology, Jining No. 1 People’s Hospital, Jining, Shandong, China; c Centre of Expertise in Mycology of Radboud University Medical Centre/Canisius Wilhelmina Hospital, Nijmegen, the Netherlands; d Department of Microbiology & Immunology, Georgetown University Medical Center, Washington, DC, USA; e Institute of Immunology and Molecular Medicine, Basic Medical School, Jining Medical University, Jining, Shandong, China; f Department of Dermatology, Jining No. 1 People’s Hospital, Jining, Shandong, China; University of Molise

**Keywords:** dandruff, hyphae, *Malassezia* spp., seborrheic dermatitis

## Abstract

Seborrheic dermatitis (SD) is a common, chronic, and relapsing skin disease. The roles of *Malassezia* spp. in the pathogenesis of SD are still not clear due to the lack of direct evidence for the existence of hyphae within affected skin tissues. We set out to elucidate if *Malassezia* mycelium contributes to the onset and development of SD and if *Malassezia* mycelium is correlated with the clinical severity of SD patients. We detected *Malassezia* hyphae in patients with SD using potassium hydroxide (KOH) and calcofluor white (CFW) staining. Fluorescent microscopy was performed for the analysis of fungal cell wall and morphological characteristics of *Malassezia* under CFW staining. Culture growth in modified Dixon agar was used for DNA extraction and sequencing, and *Malassezia* species were confirmed by a sequencing data BLAST search against the NCBI database. We demonstrated that *Malassezia* hyphae were positively correlated with the clinical severity of SD patients (*P *= 3.1738 × 10^−11^). All the patients responded well to antifungal treatment. There is no significant difference for species dominance across the variant groups. However, the exact molecular mechanisms of how *Malassezia* spp. affect SD need to be further explored. The results show that *Malassezia* spp. in the hyphal stage are restricted to SD patients compared with healthy controls, suggesting that the presence of *Malassezia* hyphae contributes to the pathogenesis of SD. The results highlight the importance of the antifungal therapy for the future treatment of SD patients.

**IMPORTANCE** Our results support the proposal that the hyphal form of *Malassezia* could be one of the pathogenic factors that contribute to SD, which has been previously less well studied. This clinical observation paves the way for further investigations of the molecular mechanisms of *Malassezia* hyphal pathogenicity in SD.

## INTRODUCTION

Seborrheic dermatitis (SD) is a chronic inflammatory skin disease and usually develops on body areas that are rich in sebaceous glands, such as the scalp, face, chest, back, axilla, and groin (1). Lipid-dependent *Malassezia* yeasts are components of the commensal microbiome on healthy human skin, but they have also been associated with several skin diseases, such as pityriasis versicolor, *Malassezia* folliculitis, and SD. It has been hypothesized that these diseases can be induced via either direct tissue invasion by filamentous forms or indirectly through immunological and metabolic processes promoted by the yeast form (2–4). For example, the mycelial form of Malassezia globosa is believed to play a dominant role in the pathogenesis of pityriasis versicolor (5).

The yeast form—but not the mycelial form—is often detected in lesion areas of SD (6). For the same reason, the pathogenic roles of *Malassezia* spp. on SD have not been defined yet (7). However, antifungal treatment seems to alleviate symptoms in patients with SD, which supports the notion that the presence of *Malassezia* could be a critical predisposing factor for SD pathogenesis (8). A morphological transformation from yeast to hyphae has been considered one of the key factors in many other fungal infections, such as *Candida* and *Malassezia* species (9, 10).

While we were looking for more sensitive approaches for fungal detection in clinical specimens, unexpectedly, we found using fluorescent dye that fungi in the hyphal form were present in the lesion areas of some patients with SD. The later experiments including culture and sequence analysis identified these fungi as *Malassezia* spp. Thus, we speculated that the presence of *Malassezia* spp. in the hyphal stage might be not random in SD patients as we previously believed. It is quite possible that the hyphal stage may be more pathogenic than the yeast form. To test these hypotheses, we increased the sample collection to 90 patients with SD and healthy controls to investigate the possible contribution of the hyphal form of *Malassezia* to the clinical symptoms of SD patients. We demonstrated the hyphal form of *Malassezia* in 36.7% of the SD patient group, with no detection of *Malassezia* species in healthy controls. Interestingly, antifungal treatment improving SD lesions was also parallel to the disappearance of hyphae.

## RESULTS

### Distribution of subjects’ age and gender.

Among 90 patients with SD, 33 patients (36.7%) were hyphal-positive confirmed under fluorescence microscopy. There was no significant difference in the distribution of age and gender between the hyphal positive group (HPG) and the hyphal-negative group (HNG) (see Fig. S1A and B in the supplemental material).

### The distribution of *Malassezia* species.

**FIG 1:**
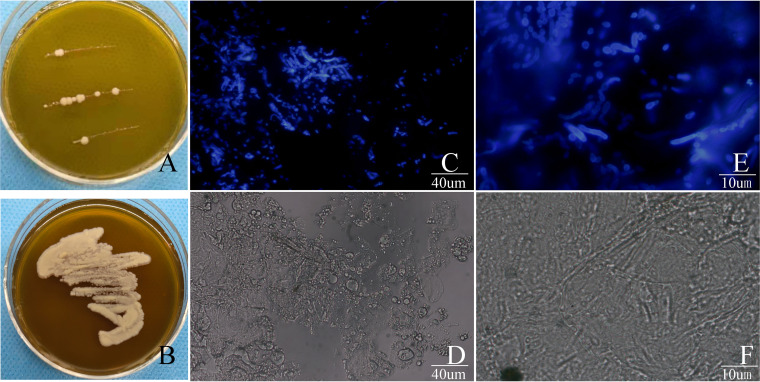
(A) Representative image of direct culture of colonies on modified Dixon agar after sampling from a skin lesion. (B) Representative image of purification of a colony of *Malassezia globosa* after culture on modified Dixon agar at 37°C for 1 week. (C) Representative image of *Malassezia* hyphae and spores stained with calcofluor white under a fluorescence microscope (original magnification, ×400). (D) The same field as in panel C under a light microscope. (E) Representative image of *Malassezia* hyphae and spores from a patient with seborrheic dermatitis stained with fluorescence stain under a fluorescence microscope (original magnification, ×1,000). (F) The same field as in panel E under a light microscope.

Fungal culture was defined as positive when at least 1 colony was observed in modified Dixon (mDixon) agar plates after 2 weeks. The colonies display cream and yellowish-brown color along the inoculating lines (Fig. 1A). Colonies were purified onto a fresh mDixon agar plate for further morphological examination and molecular identification (Fig. 1B). Very few colonies could occasionally be observed in parallel SD area (SDA) growth in less than 5% of the samples, which belongs to the same species of the fungi grown on mDixon agar confirmed by molecular sequencing.

**FIG 2:**
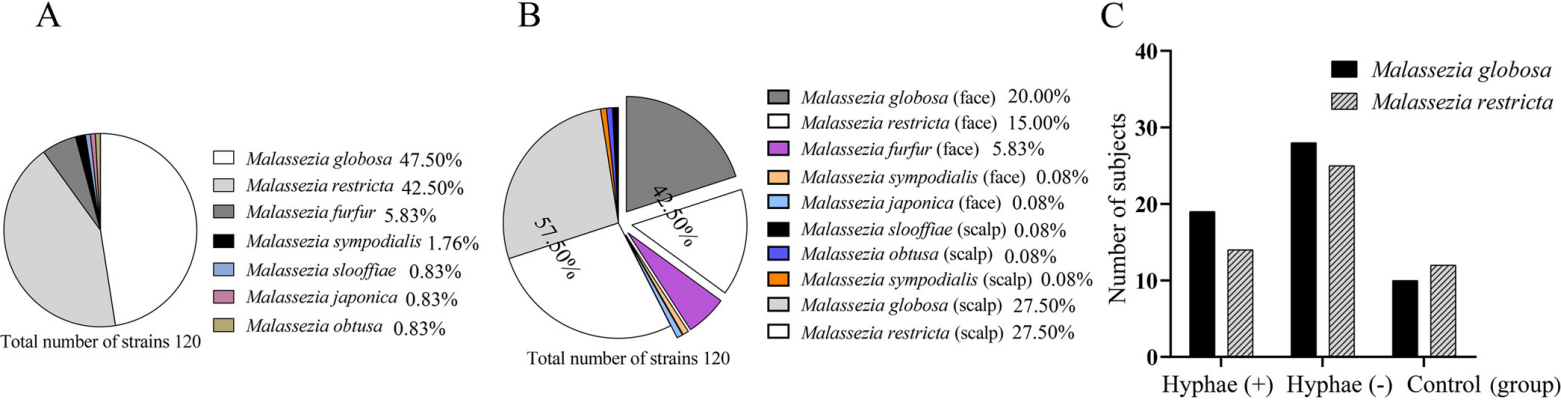
(A) Distribution of *Malassezia* species isolated in three groups. (B) Distribution of *Malassezia* spp. in lesion sites. (C) The distribution of the two predominant species (*M. globosa* and *M. restricta*) in each group (*P *= 0.566 > 0.05).

A total of 125 strains of *Malassezia* were isolated from the SD group and controls. Of these, the two predominant species were identified as *M. globosa* (62/125) and M. restricta (50/125), accounting for 89.6% of all isolates (Fig. 2A). *M. globosa* and *M. restricta* were also the most predominant species on the scalp and face. A few other species, such as M. furfur and M. japonica, were isolated only from the face, while M. slooffiae and M. obtuse were isolated only from the scalp (Fig. 2B). No significant difference was found between the HPG and HNG in the distribution of the above-mentioned 2 major species in each group by chi-square test (*P *= 0.566 > 0.05) (Fig. 2C). Meanwhile, there was no significant variation in the dominant species of gender distribution with either the HPG or HNG group (*P *= 0.247 > 0.05). Of note, we observed that 2 *Malassezia* species were isolated in 1 affected location (facial area or scalp) in 5 cases in the HPG but only 1case in the HNG. Conformation assays revealed that one of the *Malassezia* species is *M. globosa*, and the remaining are other *Malassezia* species. Among other differences, we isolated both *M. furfur* and M. sympodialis in the HPG and HNG, but in the Healthy control group (HCG); however, we identified one case of *M. slooffiae* and one case of *M. obtuse* in the HCG group.

### Clinical features and the SD area severity index (SDASI) score.

**FIG 3:**
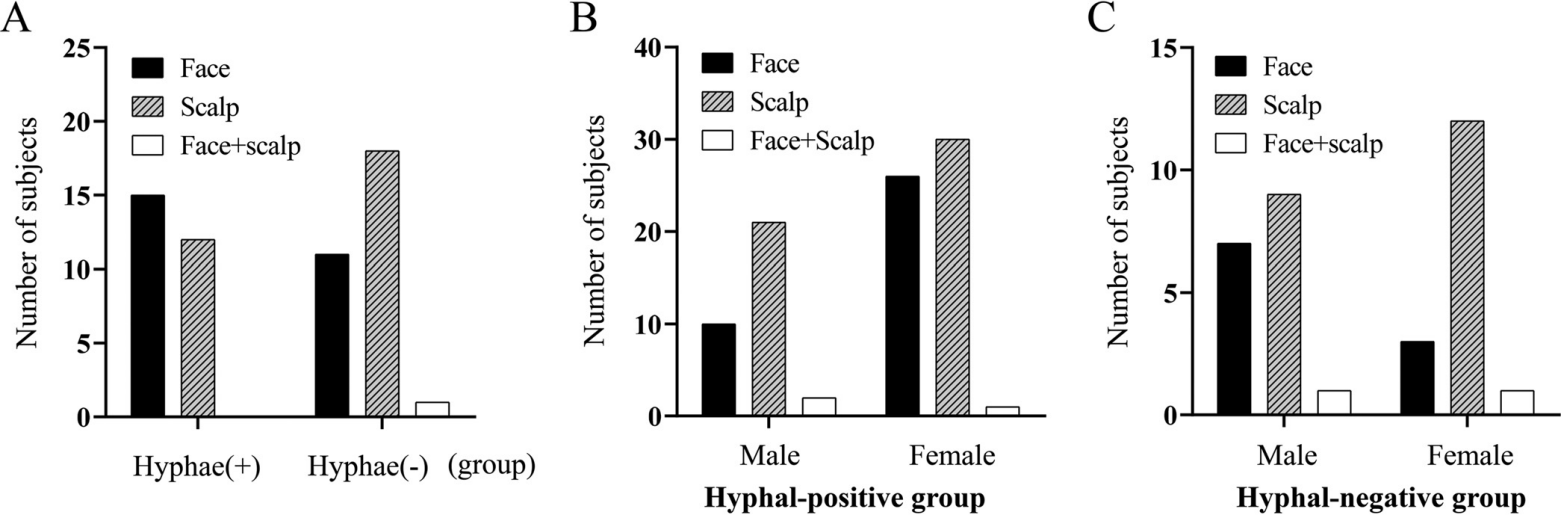
(A) Distribution of the disease sites between the hyphal-positive group and the hyphal-negative group. (B) Gender composition of the disease sites in the hyphal-positive group. (C) Gender composition of the disease sites in the hyphal-negative group.

A small percentage of the cases (3/123) had both facial and scalp involvement in both the HPG and HNG groups (Fig. 3A). There was no significant difference in the distribution of disease sites (*P *= 0.214 > 0.05) between the HPG and HNG groups (Fig. 3A). The disease sites showed no significant difference in the gender distribution of HPG and HNG groups (*P *= 0.250 > 0.05) (Fig. 3B and C).

**FIG 4:**
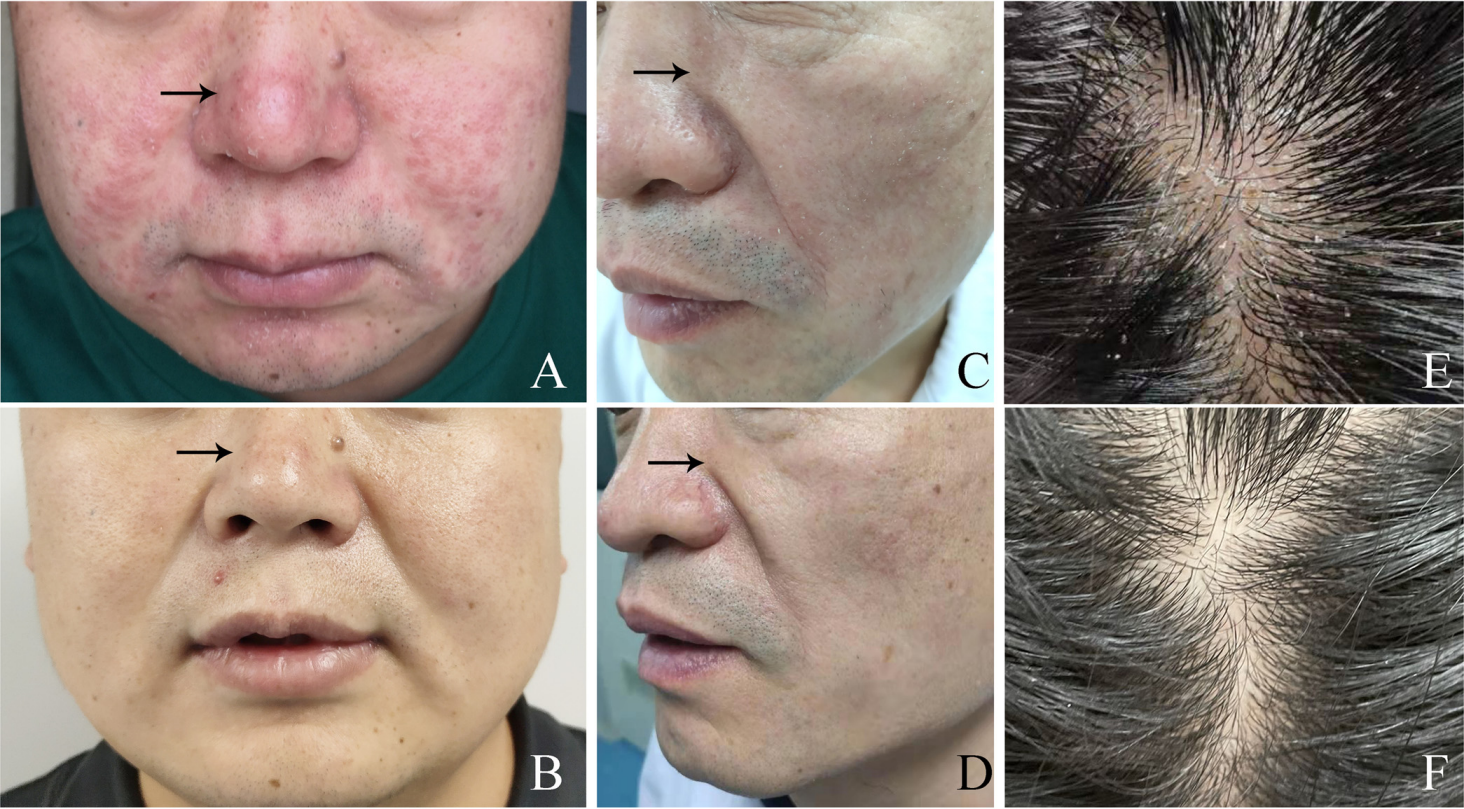
(A and B) Images showing the skin lesions on the face of a patient with seborrheic dermatitis before and after treatment, respectively. (C and D) Images showing the skin lesions on the face of a patient with seborrheic dermatitis before and after treatment, respectively. (E and F) Images showing the skin lesions on the scalp of a patient with seborrheic dermatitis.

Typically, erythema, small rashes, and sebum discharge on facial lesions appeared more obviously than on the scalp. However, the lesions showed no clear boundaries, especially if the lesions were covered with greasy material (Fig. 4A and C). All the patients reported slight itching and prolonged greasy erythema on the scalp with or without tiny dandruff (Fig. 4E).

**FIG 5:**
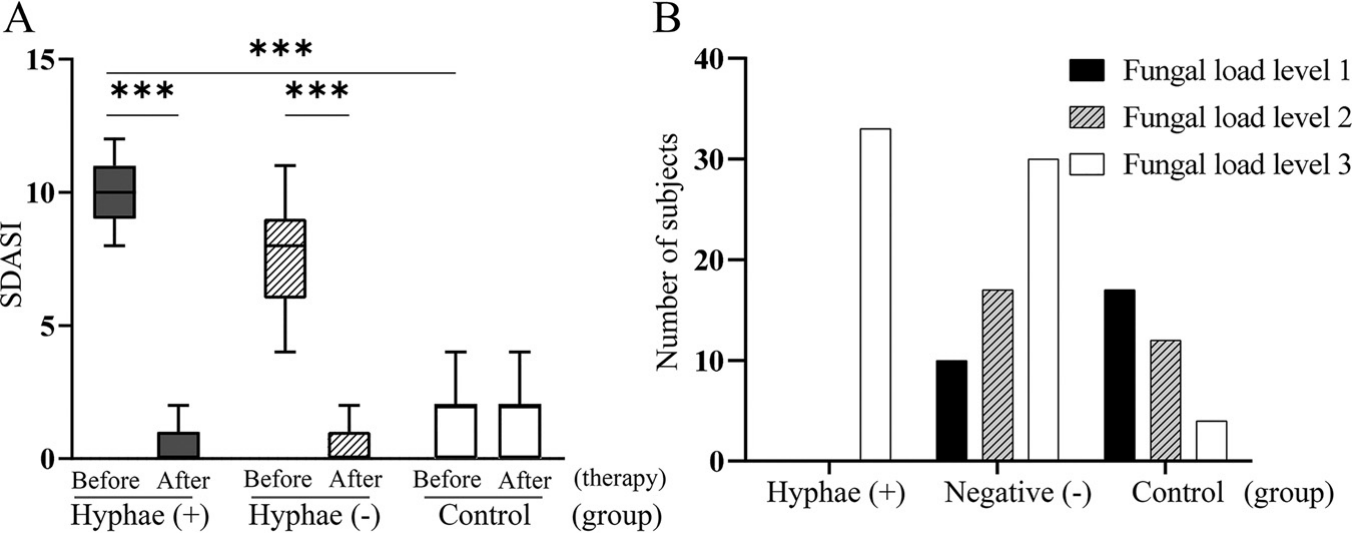
(A) The seborrheic dermatitis area severity index (SDASI) in three groups before (day 0) or after (day 28) treatment. (B) The fungal load levels (yeast plus hyphae) distributed in three groups.

The SDASI scores in both the HPG and HNG were significantly higher than those in healthy volunteers before treatment (*P *= 7.6021 × 10^−40^, *P *= 8.9922 × 10^−29^, and *P *< 0.05, respectively) (Fig. 5A); in addition, the SDASI score in the HPG was significantly higher than that in the HNG before treatment (*P *= 3.1738 × 10^−11^ and *P *< 0.05, respectively) (Fig. 5A). However, there was no significant difference in SDASI score among three groups after antifungal treatment (*P *= 0.248, *P *= 0.073, *P *= 0.078, and *P *> 0.05, respectively) (Fig. 5A). The results showed that antifungal treatment is effective for SD (Fig. 4B, D, and F), suggesting that *Malassezia* species are important for the onset of SD.

### SD patients exhibit more fungal load of *Malassezia* than healthy controls.

To quantify the fungal load in all study subjects, we performed the CFW method confirmed by the gold standard fungal culture. The results showed that CFW reached 95.1% sensitivity, and the KOH method had significantly lower sensitivity (26.3%) than fungal culture as summarized in Table 1 (Fig. 1C to F).

**TABLE 1:** Detection rate, sensitivity, specificity, and positive and negative predictive values of two microscopic examinations

Diagnostic method	Detection rate (%)	Sensitivity (%)	Specificity (%)	Positive predictive value (%)	Negative predictive value (%)
KOH	24.4	26.3	66.7	90.9	6.7
Fluorescent staining	96	95.1	60	98.2	33.3

**FIG 6:**
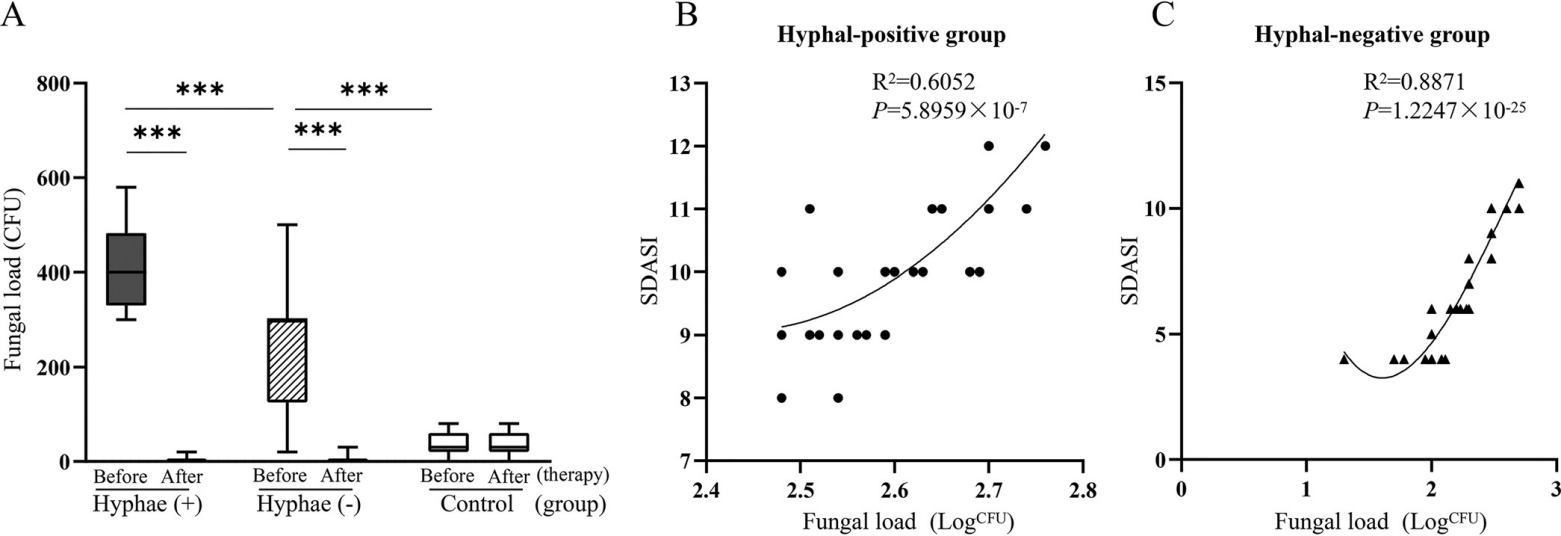
(A) The quantity of *Malassezia* yeast cells and/or hyphae in three groups before (day 0) and after (day 28) treatment. (B) Correlation curve between the quantity of *Malassezia* yeast cells and/or hyphae and SDASI in hyphal-positive groups before treatment. (C) Correlation curve between the quantity of *Malassezia* yeast cells and/or hyphae and SDASI score hyphal-negative groups before treatment. ***, *P *< 0.001 indicates statistical significance regarding the quantity of *Malassezia* yeast cells and/or hyphae (SDASI score in each group before and after treatment). SDASI, seborrheic dermatitis area severity index.

As shown in Fig. 5B and 6, before treatment the quantity of yeast cells and/or hyphal cells in samples all reached level 3 (100%) in the HPG. However, in the HNG only 52.6% of patient’s fungal loads reached level 3, and 29.8% reached level 2. Compared with the HPG or HNG, the amount of yeast cells in the HCG were more frequently on level 1 (51.5%), followed by level 2 (36.4%). In addition, the fungal load of the HPG was significantly higher than that of the HNG (*P* < 0.01) (Fig. 6A).

The results showed that there was a positive correlation between the quantity of *Malassezia* yeast cells and/or hyphae and SDASI in the HPG and HNG before treatment (HPG, *R*^2^ = 0.6525 and *P* < 0.01; HNG, *R*^2^ = 0.8871 and *P* < 0.01) (Fig. 6B and C). Thus, the results indicated that fungal load is positively correlated with the severity of SD.

### The antifungal treatment is effective for patients with high fungal load of *Malassezia*.

All 33 *Malassezia* hyphal-positive patients were treated with antifungal therapy for 4 weeks. The SDASI score decreased markedly in both the HPG and HNG after antifungal treatment compared with before treatment (*P *= 6.6203 × 10^−31^, *P *< 0.01; *P *= 4.7396 × 10^−33^, *P *< 0.01) (Fig. 5A). The lesions subsided or disappeared completely (Fig. 4 B, D, and F), and the quantity of *Malassezia* yeast cells and/or hyphae also decreased markedly in both the HPG and HNG after antifungal treatment (*P *= 3.7876 × 10^−25^, *P *< 0.01; *P *= 7.1127 × 10^−22^, *P *< 0.01) (Fig. 6A).

The correlation between SDASI and fungal loads was plotted for the HPG or HNG groups (Fig. 6B and C). As shown in Fig. 6B and C, the severity of the disease (SDASI) is positively correlated with fungal loads in both the HPG (*R*^2^ = 0.6025; *P* = 5.8959 × 10^−7^) and HNG (*R*^2^ = 0.8871; *P* = 1.2247 × 10^−25^). The results suggest that the reduction of *Malassezia* hyphal and/or yeast loads by antifungal treatment contributes to the remission of clinical symptoms of SD patients.

## DISCUSSION

*Malassezia* is the most prevalent fungal genus on healthy skin (11), and this group of lipid-dependent fungi utilizes exogenous fatty acids for growth due to their lack of fatty acid synthase genes (12). *Malassezia* poses a significant pathogenic potential under certain conditions (13). *Malassezia* spp. are predominantly found on lipid-rich anatomic areas such as the face, scalp, and trunk, which are also commonly affected areas in SD patients (14). Thus, it is generally believed that the genus *Malassezia* is involved in the pathogenesis of SD, but there is no solid published evidence. The current study aimed to investigate the casual relationship between the presence of *Malassezia* and the pathogenesis of SD.

Since there was no evidence of hyphae in most SD conditions, the potential mechanism for *Malassezia* to trigger disease conditions lies in the regulation of immunological or metabolic processes by yeast cells (2, 12). Interestingly, we observed hyphal presence in more than one-third of patients with SD in this clinical study. Furthermore, we demonstrated that hyphal-positive patients manifested more severe clinical symptoms based on SDASI score and, in parallel, have higher levels of fungal loads detected from their lesions. Similar to the previous report that antifungal therapy targeting the *Malassezia* yeast form was effective in SD patients (12), unexpectedly, we found that short-term antifungal treatment relieved clinical symptoms, constrained fungal loads, and led to disappearance of hyphae in these hyphae-positive SD patients. Given the fact that the hyphae disappeared or decreased in conjunction with remission or disappearance of lesions, we believe that *Malassezia* hyphae could be one pathogenic factor of SD. Paradoxically, topical treatment also significantly reduces the fungal load in the HNG group, suggesting that the nonhyphal fungal load could be another reason for the clinical severity of SD. Thus, the findings in the present study support the notion that the transition of *Malassezia* from yeast to hyphae could enhance virulence and play a critical role in the pathogenesis of SD, reminiscent of the ones found in other yeast species bearing a greater virulence potential than the yeast form. The exact importance of the *Malassezia* hyphal or yeast form in the pathogenesis of SD requires further investigation in the future.

In the present study, the enrolled SD patients were mainly composed of adolescents and adults from our dermatology outpatients. The age of SD patients clustered between 13 and 39 years-old, consistent with the peak in sebum gland activity. The fact that SD occurs only in seborrheic areas raises the question of whether these incidence peaks correlate with defined environmental, *Malassezia* and/or hormonal (e.g., androgen-based) changes in the skin milieu (15). Sebum is secreted by hormonal stimulation, and its secretory activity increases rapidly at the onset of adolescence, remaining stable through the 20s and 30s, and then gradually decreases with increasing age. In females, sebum secretion decreases rapidly after menopause, but in males, the secretion rate can be maintained at high levels until 50 or 60 years of age (16). While both hyphal-negative and hyphal-positive patients with SD fall in the same age group for high sebum secretion activity, there were 3 hyphal-positive male patients over 50 years old in this study, which could be due to a higher SG activity. Although SD is two times more common in males than in females (17), there was no gender imbalance in our SD patients. We believe that women usually pay more attention than men to their appearance and thus were more likely to seek medical advice.

The common *Malassezia* spp. that cause human dermatosis are *M. globosa*, *M. restricta*, *M. sympodialis*, and *M. furfur.* In western countries, *M. restricta* and *M. globosa* are more common than other species in cases of SD (18). In the present study, our results are reproducible with a previous study finding that *M. globosa* and *M. restricta* are predominant in SD patients. The distinct appearance of *M. globosa* in pityriasis versicolor has been described as “spaghetti and meatballs” for short hyphal structures and abundant yeasts (19), which was observed in our hyphal-positive samples (Fig. 1C). In fact, we detected 4 cases of *M. furfur*-caused facial SD in the HNG group and 3 cases in the HPG group. We believe that the hyphal formation of *Malassezia* is not necessarily species-specific and is likely a response to microenvironmental factors.

Until recent decades, laboratory diagnostic procedures in dermatological mycology have depended on direct microscopy with application of potassium hydroxide (KOH) solution 10% to 30% (20). Within this concentration range, KOH is a keratin-digesting reagent that dissolves proteins, lipids, and epithelial cells, while the fungal components on the cell walls (i.e., chitin and glycoproteins) resist the digestion of KOH. Calcofluor white (CFW) is a fluorescent stain that recognizes chitin, cellulose, and other β(1-3) and β(1, 4)-linked polysaccharides. With easily accessible long-wavelength UV and short-wavelength visible light (21), CFW staining is increasingly being used clinically for its high sensitivity in detecting these fungi. The CFW and KOH used in this study also showed a greater sensitivity of CFW than simple KOH light microscopy. Under light microscopy, it was difficult to distinguish *Malassezia* elements from host keratin cells in most cases (Fig. 1D and F), with high false-positive and false-negative rates by the KOH method. In contrast, it was easier to distinguish *Malassezia* yeast, budding yeasts, or short hyphae from a clean background by CFW staining even when *Malassezia* elements were scarce in the samples (Fig. 1C and E). Undoubtedly, the sensitive CFW method has clear advantages in detection and quantification of *Malassezia*. As fungal fluorescent staining had not been widely applied for *Malassezia* detection in sebum skin samples in clinical settings, we thought that this could be the reason for not detecting *Malassezia* hyphae in SD patients.

In conclusion, our results support the proposal that the hyphal form of *Malassezia* could be one of the pathogenic factors that contribute to SD, which has been previously less studied. This clinical observation paves the way for further investigations of the molecular mechanisms of *Malassezia* hyphal pathogenicity in SD.

## MATERIALS AND METHODS

### Study subjects.

**(i) Selection criteria of patients with SD.** The diagnosis of SD is based on clinical features of red, flaky, greasy areas appearing on the scalp, nasolabial folds, ears, eyebrows, and chest, accompanied by itching or hair loss. The SD area severity indexes (SDASIs) are scored as 0 (none), 1 (mild), 2 (moderate), and 3 (severe) by areas and severity of erythema, scaling, and pruritic itching in scalp and facial areas, including forehead/eyebrows, paranasal, chin/perioral, and ears (22). The SDASI scores were evaluated before and after treatment (at days 0 and 28).

**(ii) Exclusion criteria.** All of the subjects had no concurrent skin inflammatory disorders, pityriasis versicolor, or other disease history. Patients or healthy volunteers with previous topical antifungal and/or steroid treatment for SD or dandruff within 1 month prior to sampling were excluded.

The study enrolled 90 patients diagnosed with SD and 33 healthy individuals as controls. All of the subjects were recruited from 1 August 2020 to 31 January 2021 in Jining No. 1 People’s Hospital. The healthy individuals in the control group matched the hyphal-positive patients by age, gender, and skin sampling area. The study was approved by the Ethics Committee of Jining No. 1 People’s Hospital. The samples were collected after informed-consent documents were agreed on by the subjects.

### Specimen collection and fungus load assessment.

Flakes or scales were scraped from the scalp of SD patients or healthy individuals within a 1-inch square area using a sterile blunt scalpel after parting the hair with a sterile comb. For patients with facial SD presenting skin lesions on the nose surface, the second sample for each subject was collected in parallel from the nasolabial folds using a sterile blunt scalpel. Half of the scraped dandruff or skin material collected from 1 cm^2^ of the lesion area or healthy skin of volunteers was submerged in a drop of 5 μl fungal fluorescence stain (Jiangsu Medomics Medical Technology Co., Ltd., Nanjing, China) prior to placement on the slide. After the slide was covered, the staining required a further 5 min for full staining, and fungal yeasts and hyphae were examined under fluorescence microscopy.

The whole slide was examined carefully under a fluorescence microscope, and 5 magnification fields (at ×400) were randomly selected to quantify yeast cells or hyphae. The average fungal populations per field were compared, and the amounts of *Malassezia* cells per unit skin area were categorized as level 1 if fewer than 100, level 2 if 100 to 300, and level 3 if more than 300. Additionally, the patients were placed into the hyphal-positive group (HPG) whenever the hyphal cells were greater than 10 events in any magnification field (×400) and otherwise into the HNG.

### Isolation and identification of *Malassezia*.

The second half of each specimen from the lesion areas or healthy controls was divided into two parts. One part was inoculated onto mDixon agar plates (Hopebio, Qingdao, China), and another part was inoculated onto Sabouraud glucose medium (SDA) and then incubated at 37°C for 2 weeks. The colonies formed in mDixon agar but not in SDA were reexamined with fluorescent staining and were used for molecular identification next. These were also tagged for extraction of fungal DNA for sequencing.

Fungal DNA extraction and sequencing from colonies resembling *Malassezia* by phenotypic characteristics under microscopy were performed. Genomic DNA was extracted from these purified colonies with a fungal DNA kit (Qiagen, Germantown, MD, USA) according to the manufacturer’s instructions. DNA extractions were analyzed directly or stored at –20°C for further use. Amplification was performed using the primers ITS1 (5-TCCGTAGGTGAACCTGCGG-3′) and ITS4 (5-TCCTCCGCTTATTGATATGC-3′). The sequences obtained were aligned with MEGA v5, and species identification was carried out using online sources of the GenBank and Westerdijk Fungal Biodiversity Centre databases.

### Treatment.

The HPG patients were treated with oral itraconazole at 0.2 g per day in combination with topical 2% ketoconazole cream for 2 weeks. The HNG patients received 2% ketoconazole cream only. The SDSCI score was evaluated before and after treatment with antifungal agents.

### Statistical analysis.

SPSS v23 was used for all analysis in the present study. Chi-square analysis was performed to determine the difference in gender between the patient group and the healthy group and between patients with and without hyphae. The independent sample *t* test was used to detect the difference in age distribution between the HPG and HNG. The rank sum test was used to examine the distribution of spore numbers between three groups. A two-tailed *P* value of <0.05 was regarded as statistically significant. Taking the positive culture as the gold standard, the sensitivity and specificity of the two microscopic examination methods were evaluated at the same time.

### Data availability.

All of the sequence data were deposited in GenBank (accession numbers OL691075, OL691076, and OL691790 to OL691907).
