# Supplementary material for: Presence of Malassezia Hyphae Is Correlated with Pathogenesis of Seborrheic Dermatitis
Source: Microbiol Spectr. 2022 Jan 12;10(1):e01169-21. doi: 10.1128/spectrum.01169-21 (PMC8754105; doi:10.1128/spectrum.01169-21)
Supplement: SUPPLEMENTAL FILE 1 — Supplemental material. Download SPECTRUM01169-21_Supp_1_seq11.pdf, PDF file, 0.2 MB [file spectrum01169-21_supp_1_seq11.pdf]

**Supplemental material**

Presence of *Malassezia* hyphae is correlated with pathogenesis of seborrheic dermatitis

Juanjuan Li[1#], Yahui Feng[1#], Chen Liu[2], Zhiya Yang[2], Sybren de Hoog[3], Yuying Qu[1], Biao Chen[2], Dongmei Li[4], Huabao Xiong[5*], Dongmei Shi[2,6*]

[1]Department of Clinical Medicine, Jining Medical University, Jining 272067, Shandong, China
[2]Laboratory of Medical Mycology, Jining No. 1 People's Hospital, Jining 272067, Shandong, China
[3]Centre of Expertise in Mycology of Radboud University Medical Centre / Canisius Wilhelmina Hospital, 6525, Nijmegen , The Netherlands
[4]Department of Microbiology & Immunology, Georgetown University Medical Center, Washington DC 20057, USA
[5]Institute of Immunology and Molecular Medicine, Basic Medical School, Jining Medical University, Jining 272067, China.
[6]Department of Dermatology, Jining No.1 People's Hospital, Jining 272067, Shandong, China

[#] The authors contributed to this article equally.

Corresponding authors:
Correspondence to: Dongmei Shi, Dermatology Department of the First People's Hospital of Jining, Shandong, 272011, China. Phone: +86 537-6051008; Email: shidongmei28@163.com, or to Huabao Xiong, Institute of Immunology and Molecular Medicine, Jining Medical University, Jining, Shandong, 272067, China, Phone: +86 537-3616283; Email: xionghbl@yahoo.com

Supplemental Fig. 1

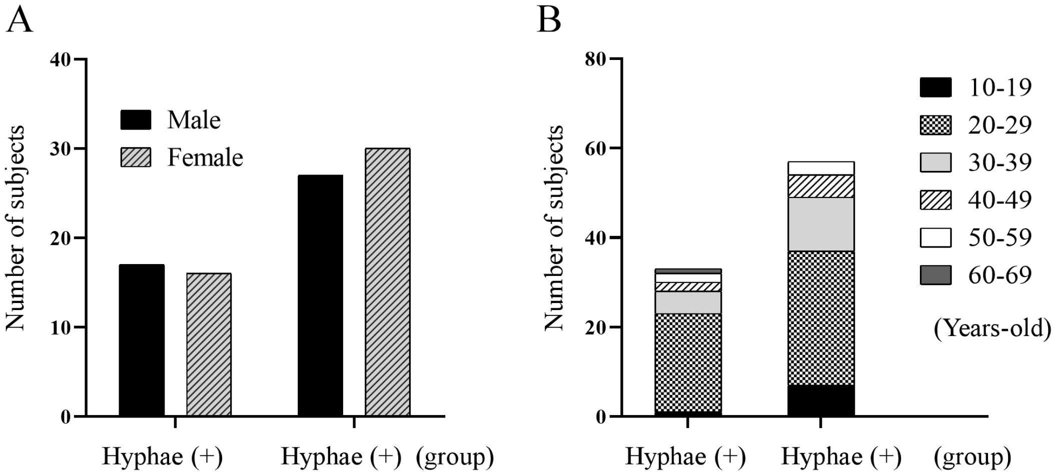


Supplemental Figure 1. A: General information of Hyphae-positive groups and Hyphae- negative groups. B: Age distribution of Hyphae-positive group and Hyphae-negative group.
